# Altered Hemodynamic Activity in Conduct Disorder: A Resting-State fMRI Investigation

**DOI:** 10.1371/journal.pone.0122750

**Published:** 2015-03-27

**Authors:** Jiansong Zhou, Nailin Yao, Graeme Fairchild, Yingdong Zhang, Xiaoping Wang

**Affiliations:** 1 Mental Health Institute of The Second Xiangya Hospital, Central South University; Hunan Province Technology Institute of Psychiatry; Key Laboratory of Psychiatry and Mental Health of Hunan Province, Changsha, China; 2 Department of Psychiatry, The University of Hong Kong, Hong Kong, China; 3 Academic Unit of Psychology, University of Southampton, Southampton, United Kingdom; University of Maryland, College Park, UNITED STATES

## Abstract

**Background:**

Youth with conduct disorder (CD) not only inflict serious physical and psychological harm on others, but are also at greatly increased risk of sustaining injuries, developing depression or substance abuse, and engaging in criminal behaviors. The underlying neurobiological basis of CD remains unclear.

**Objective:**

The present study investigated whether participants with CD have altered hemodynamic activity under resting-state conditions.

**Methods:**

Eighteen medication-naïve boys with CD and 18 age- and sex- matched typically developing (TD) controls underwent functional magnetic resonance imaging (MRI) scans in the resting state. The amplitude of low-frequency fluctuations (ALFF) was measured and compared between the CD and TD groups.

**Results:**

Compared with the TD participants, the CD participants showed lower ALFF in the bilateral amygdala/parahippocampus, right lingual gyrus, left cuneus and right insula. Higher ALFF was observed in the right fusiform gyrus and right thalamus in the CD participants compared to the TD group.

**Conclusions:**

Youth with CD displayed widespread functional abnormalities in emotion-related and visual cortical regions in the resting state. These results suggest that deficits in the intrinsic activity of resting state networks may contribute to the etiology of CD.

## Introduction

Conduct disorder (CD) is characterized by a persistent pattern of antisocial behavior and aggression in childhood and adolescence [1]. Youth with CD not only inflict serious physical and psychological harm on others, but are at increased risk of sustaining personal injuries, as well as developing depression or substance abuse, and participating in recurrent criminal behaviors in adulthood [2,3]. The underlying neurobiology of CD remains unclear but has attracted growing attention in recent years [4].

Adolescents with CD showed reduced gray matter volume (GMV) in diverse cortical regions when compared to typically-developing (TD) comparison subjects in previous studies [5,6]. In addition, reduced neural responses have also been reported in diverse areas including the amygdala, anterior cingulate cortex, insula, and orbitofrontal cortex in youth with CD compared to TD controls [7–10]. Although most of these functional magnetic resonance imaging (fMRI) studies reported alterations in emotion- and cognition-related cortical and subcortical areas in CD [8], the direction of the effects differed between studies. For example, Sterzer et al. (2005) found reduced amygdala responses to negative pictures in adolescents with CD [11] whereas Herpertz et al. (2008) reported increased amygdala responses to negative pictures in CD [12]. This discrepancy in fMRI findings could be due to differences in task design across the studies. For this reason, an investigation of brain activity under baseline conditions in adolescents with CD would be informative and could have clinical and prognostic value.

Spontaneous low-frequency (<0.08 Hz) fluctuations of the blood oxygen level-dependent (BOLD) signal in the brain (as measured using resting-state functional magnetic resonance imaging, rs-fMRI) [13] have been shown to be closely related to spontaneous neuronal activity [14,15]. These fluctuations are believed to reflect baseline activity that is largely unrelated to cognitive activity [16]. A method that involves assessing amplitude of low-frequency fluctuations (ALFF) has recently been developed by Zang et al. [17] to measure whole-brain resting-state (rs)-fMRI. ALFF has been linked to neuronal glucose metabolism [18] and correlates with local field potential activity [19]. Alterations in ALFF have described in a number of disorders including schizophrenia and major depressive disorder [20,21], making the patterns of ALFF alterations potentially useful biomarkers for complex neuropsychiatric conditions. Therefore, this method could be used to assess the activity of intrinsic brain networks in patients with CD to examine whether they show abnormalities in resting state activity.

CD is characterized by deficits in guilt and empathy, impaired recognition of emotions [22–24], and impulsive, risky decision-making [22,25]. As these traits have been linked to emotion regulation centers such as the amygdala, insula, and anterior cingulate cortex (ACC) [26–30], and given previous evidence for changes in task-related activity in these regions [7–12], we hypothesized that participants with CD would show altered ALFF in these regions. If this hypothesis was supported, this might help to explain why CD is associated with deficits in emotion processing and decision-making.

A significant methodological advantage of our study is that we deliberately recruited a pure CD group by excluding participants who had comorbid attention-deficit/hyperactivity disorder (ADHD) or substance use disorders, which might account for some of the discrepancies in the results of previous studies of CD [31], although these confounding effects have been statistically controlled for in some studies [32–34]. Thus, studying a more homogenous sample of CD patients could elucidate the specific neurobiological factors underlying this developmental disorder. Finally, given that there are sex differences in the prevalence and developmental course of CD [35–38], this study focused on young male subjects only.

## Methods and Materials

### Participants

Thirty-six boys participated in this study, 18 with a DSM-IV diagnosis of CD and 18 age-matched typically-developing (TD) controls. All subjects were right-handed, and aged between 15 and 17 years. Participants with CD were recruited from the Hunan province Youth Detention Centre (YDC) in the People’s Republic of China, whereas the participants in the TD group were recruited from schools in the community of Changsha, Hunan province. Information sheets describing the aims, content, and duration of the study were given to all the participants and parents, and all participants and parents provided written informed consent. This study was approved by the Biomedical Ethics Board of the second Xiangya Hospital, Central South University, People’s Republic of China (S20080082).

Current and lifetime psychiatric problems were assessed in both CD and TD subjects by an experienced child psychiatrist using the Chinese version of the Schedule for Affective Disorder and Schizophrenia for School-Age Children-Present and Lifetime (K-SADS-PL) [39–41]. This is a semi-structured psychiatric interview based on DSM-IV criteria (American Psychiatric Association, 1994). The K-SADS-PL includes: 1) an unstructured Introductory Interview; 2) a Diagnostic Screening Interview; 3) the Supplement Completion Checklist; 4) the appropriate Diagnostic Supplements; 5) the Summary Lifetime Diagnoses Checklist; and 6) the Children's Global Assessment Scale (C-GAS) ratings. Participants with a history of neurological disorders including paralysis, loss of sensation, muscular weakness, epilepsy, seizures, chronic pain, confusion, and prolonged loss of consciousness due to head injury were excluded from the study. The majority of the items are scored using a 0–3 point rating scale (0: no information is available, 1: the symptom is not present, 2: subthreshold levels of symptomatology, and 3: threshold criteria). Any participants meeting the K-SADS-PL criteria for any other current or lifetime psychiatric disorder except CD, such as ADHD, mood disorder, anxiety disorder, mental retardation, or substance abuse or dependence were also excluded from the study.

### Psychological assessment

The self-report questionnaire Screen for Child Anxiety Related Emotional Disorders (SCARED) [42] was used to assess anxiety disorder symptoms. The Birleson Depression Self-Rating Scale (DSRS) was used to assess depressive symptoms experienced during the preceding week [43]. Further details regarding the SCARED and DSRS questionnaires were reported in our previous articles [44,45].

### MRI data acquisition

MRI data were obtained with a 3-Tesla scanner (Siemens Allegra; based at the Magnetic Resonance Center of Hunan Provincial People’s Hospital, People’s Republic of China) using an 8-channel phased-array head coil with participants lying in a supine position. Blood oxygen level-dependent (BOLD) functional MRI data were acquired using a gradient-echo echo-planar imaging (EPI) sequence [46,47]. Acquisition parameters were as follows: repetition time (TR) = 3000 ms, echo time (TE) = 30 ms, flip angle = 90°, 100 volumes, 36 contiguous axial slices, anterior-posterior acquisition, in-plane resolution = 3.75 × 3.75 × 3.75 mm, slice thickness = 3mm, and field of view = 256×256mm. The overall acquisition time was = 6 minutes and 36 seconds. Slice acquisition order was contiguous. To reduce scanner noise and head motion, a foam pillow, extendable padded head clamps, and ear plugs were used when participants were in the scanner. Participants were asked to simply rest in the scanner remaining awake and alert, with their eyes closed and to stay as still as possible during the resting state scan. Three-dimensional T1-weighted anatomical MRI data were acquired with a fast field echo sequence (Magnetization Prepared Rapid Gradient Echo, MPRAGE), using the parameters TR = 2000ms, TE = 3.36 ms, flip angle = 9°, 1 × 1 × 1mm voxels, FOV = 256×256mm, number of slices = 144.

### Functional data analysis

#### Image preprocessing of resting state fMRI data

Preprocessing of resting state fMRI data was performed using SPM8 (http://www.fil.ion.ucl.ac.uk/spm) software. The first 10 volumes were discarded for each individual to allow for the effects of magnetic saturation. All functional images were corrected for slice timing and head movement. All 36 participants remained within < 2 mm of displacement in any direction and within 2 degrees of rotation in any direction, so none of the data were excluded from further analysis due to excessive head movement. Prior to band-pass filtering (0.01∼0.08Hz) which controls for the physiological "noise" (cardiac and respiratory-related artifacts), the following nuisance covariates were regressed from the BOLD signal [48]: 6 rigid-body parameters, white matter signal, and cerebrospinal fluid (CSF) signal. After band-pass filtering, all functional data were normalized to the Montreal Neurological Institute (MNI) space by applying the transformation parameters obtained from the structural images (see the following “structural image analysis” section for details) and smoothed (4 mm full width at half maximum (FWHM) Gaussian kernel).

#### ALFF calculations

ALFF calculations were performed using REST software version 1.8 (www.restfmri.net) [49]. First, for each voxel of the brain, the BOLD time series was converted to the frequency domain using a Fast Fourier Transform. The square root of the power spectrum was then calculated and averaged across a specified frequency range (0.01–0.08Hz) to eliminate remaining low- and high-frequency noise in the resting state data [13,50]. This value was then transformed using Fisher’s Z and is referred to as the ALFF for a given voxel [17].

#### Structural image analysis

Individual structural T1-weighted images were co-registered to the mean motion-corrected functional images using a linear transformation. They were then segmented into grey matter (GM), white matter (WM), and cerebrospinal fluid (CSF) in MNI space by using "New Segment" in SPM8. The DARTEL procedure [21] was then used to create a study-specific template. GM, WM and CSF were then normalized to MNI space and smoothed with an 8 mm FWHM Gaussian kernel. Mean modulated and smoothed GM maps (intensity threshold = 0.2) were used to generate a group GM mask and applied as a mask for analyzing ALFF differences in the comparisons, specific to the groups included in a particular test.

### Statistical analysis

Demographic factors were compared using independent two-sample t-tests using the Statistical Package for Social Sciences (version 15.0.1) (SPSS for Windows, 2006) [51]. Between-group voxel-wise comparisons were performed using non-parametric permutation tests (5000 permutation) implemented in RANDOMISE [52] in FSL. Statistical comparisons between groups in terms of ALFF were restricted to regions within the corresponding GM mask generated as described above. The voxel-based statistical tests were corrected for multiple comparisons at a significance level of *p*<0.05 using Monte Carlo simulations (uncorrected single voxel significance level of *p*<0.001 and a minimum cluster size based on the size of the gray matter mask) [53,54]. The relationship between the number of CD symptoms in the K-SADS-PL and ALFF was assessed within the CD group using Pearson’s correlations.

## Results

### Demographic and clinical characteristics

The CD and TD groups were matched in terms of age, education level, parental education levels, parental marital status, family income, and levels of self-reported anxiety and depression (Table 1).

**Table 1:** Demographic characteristics and descriptive statistics for the CD and TD.

	CD (*n* = 18)		TD (*n* = 18)			*df*	*p*
	*n*	*%*	*n*	*%*	*χ* ^*2*^		
**Parental marital status (divorced)**	4	22.2	1	5.6	2.1	1	0.15
**Family income (monthly)** ^**a**^					1.8	3	0.62
***<1000***	5	27.8	2	11.1			
***1000–1999***	7	38.8	8	44.5			
***2000–3499***	5	27.8	6	33.3			
***>3500***	1	5.6	2	11.1			
	*mean*	*sd*	*mean*	*sd*	*t*		
**Age (years)**	16.1	0.5	15.9	0.3	1.1	34	0.27
**Duration of education (years)**	9.4	2.0	9.2	1.9	0.7	34	0.47
**Father’s duration of education (years)**	8.8	2.6	10.4	2.2	1.9	34	0.07
**Mother’s duration of education (years)**	8.2	4.1	10.1	3.5	1.6	34	0.13
**K-SADS-PL**							
**Positive items of CD**	7.4	2.1	0.0	0.0	14.8	34	<0.001
**Total symptoms Score of CD**	31.6	4.1	15.7	0.7	16.1	34	<0.001
**SCARED**	16.1	7.4	17.4	10.4	0.4	32	0.67
**DSRS**	11.8	3.8	9.8	4.1	1.6	34	0.12

SCARED = Screen for Child Anxiety Related Emotional Disorders; DSRS = Birleson Depression Self-Rating Scale. K-SADS-PL: the Schedule for Affective Disorder and Schizophrenia for School-Age Children-Present and Lifetime.

^a^ Chinese RMB (yuan) minimum monthly salary (per person).

### Group differences in resting state activity

**Fig 1:**
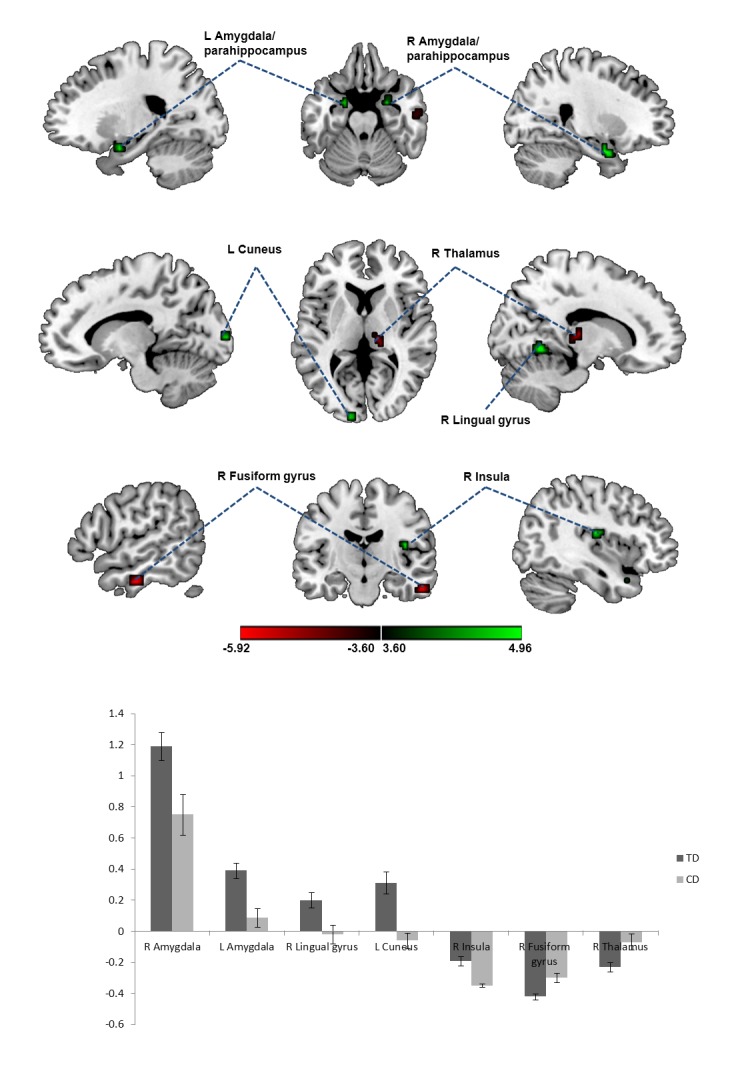
ALFF result in participants with CD compared to TD participants.

As shown in the Fig 1 and in Table 2, compared with the TD participants, the CD participants showed decreased ALFF in the bilateral amygdala/parahippocampus, right lingual gyrus, left cuneus and right insula. In contrast, the CD participants showed increased ALFF in the right fusiform gyrus, and right thalamus relative to the TD participants. There was no significant correlation between the number of CD symptoms and ALFF results in the CD group.

**Table 2:** The significant differences ALFF brain areas between CD and TD groups.

Direction of difference	Number of voxels	x	y	z	Side	Brain regions	BA	T-value
**Lower in CD**	18	25	2	-25	R	amygdala/parahippocampus	28	4.96
	7	-20	-1	-22	L	Amygdala/parahippocampus	n/a	4.94
	14	13	-58	-4	R	Lingual gyrus	19	4.81
	6	-11	-100	5	L	Cuneus	n/a	4.22
	6	40	-16	17	R	Insula	13	4.59
**Greater in CD**	16	58	-10	-28	R	Fusiform gyrus	20	-5.92
	9	13	-22	8	R	Thalamus	n/a	-4.58

Group ALFF differences are shown at p < 0.001 of multiple comparison correction (cluster size = 162 mm3, T > 3.6 (or T < −3.6)). x, y, z: coordinates in the MNI atlas extending from z = -65 mm to +80 mm. T values are from a t-test of the peak voxel (showing greatest statistical difference within a cluster); a negative T value means greater ALFF in the CD group. Both age and education were regressed from the data as nuisance covariates.

BA: Brodmann area. L, R: left and right.

Difference maps at the given threshold, corrected for multiple comparisons (Monte-Carlo Simulation, cluster size = 162 mm^3^ (6 voxels), T > 3.6 (or T < −3.6), and *p*< 0.001, uncorrected). Green indicates regions where participants with CD had lower ALFF when compared with TD participants, while red indicate regions the converse. The column bars show average ALFF z scores (±standard error) of the clusters (as listed orderly in Table) with significantdifference between TD and CD groups.

## Discussion

The current study is, to our knowledge, the first to identify functional hemodynamic changes in boys with CD relative to typically-developing controls during the resting state. The results support the hypothesis that emotion-related areas including the amygdala and insula would show altered hemodynamic fluctuations in participants with CD. The findings also revealed group differences in visual regions such as the left cuneus, right lingual gyrus and right fusiform gyrus, although increased ALFF was observed in the latter region in participants with CD relative to TD controls

The amygdala and insula are strongly implicated in emotion processing [55], previous studies reported reduced gray matter volume in the amygdala and insula [5], and reduced amygdala activation in affective tasks in male adolescents with CD [55]. This study is the first to observe decreased ALFF was revealed in CD compared to TD participants. As ALFF measures the synchronicity of neuronal activity signals among remote regions of the brain, and the ALFF signal has been shown to reflect regional spontaneous neuronal activity, the lower ALFF exhibited in the amygdala and insula in CD individuals might reflect decreased energy consumption in these regions [18]. The insula [56], amygdala, and regions in the prefrontal cortex such as the orbitofrontal cortex [57] have been identified as critical structures for emotion processing, motivation, decision-making [28,58–60], and are functionally linked to negative emotional responses [61,62] and arousal effects [63]. These results suggest that the abnormal spontaneous activity of the amygdala and insula may play a role in the underlying pathophysiology of children with CD [22,23,64].

Another interesting finding was that lower ALFF was observed in right lingual gyrus and left cuneus, whereas higher ALFF values were detected in right fusiform gyrus in CD compared to TD individuals. The cuneus is involved in basic aspects of visual processing [65]. The lingual gyrus is linked to visual processing, logical reasoning [66] and visual memory encoding [67]. Our finding of lower ALFF in lingual gyrus and cuneus is in line with previous studies showing decreased activation in lingual gyrus in women with borderline personality disorder [68]. Moreover, a recent study reported that activity in lingual gyrus and cuneus were negatively correlated with risk-taking in CD individuals [69]. The fusiform gyrus appears to be involved in facial identity processing, and plays an important role in facial expression perception [70]. Previous fMRI studies reported mixed findings in terms of the activity of the fusiform gyrus in CD [71–73] which might be due to differences between fMRI tasks in cognitive load or difficulty. Our study is the first to investigate hemodynamic functioning in adolescents with CD during the resting state, which is argued to be a better reflection of “baseline” function than task-related activity. We speculate that higher ALFF in fusiform gyrus implies greater spontaneous neuronal activity at rest [18] possibly leaving less ‘reserve’ for the demands of fMRI tasks involving emotion processing. This hypothesis could be tested in future studies using alternative methods that provide measures of brain metabolic activity such as arterial spin-labelling [74].

Finally, the present study showed increased spontaneous neuronal activity in the thalamus of CD compared with TD participants. Altered functional activity was reported in previous fMRI studies. One study showed increased thalamus activity during the Stroop task in CD patients compared to TD controls [75], while another study showed that the thalamus was more active in violent offenders than non-offenders during a conflict-related task [76]. The thalamus is thought to play an important role in stress adaptation and controlling motor systems [76]. Therefore, it is presently unclear whether the abnormal spontaneous thalamic activity observed in the present study is related to deficits in emotion processing or cognitive control. Consequently, future studies could investigate the impact of abnormal thalamus activity on performance of emotional and cognitive tasks in CD participants.

### Strength and limitations

To our knowledge, this is the first study to investigate baseline fluctuations in hemodynamic activity in individuals with CD. This pilot study will be helpful in terms of designing future studies investigating functional abnormalities in CD at the network level, based on the deficits exhibited by the present sample. These studies could use graph theoretical methods to explore patterns of connectivity during the resting state. It would also be interesting to investigate structure-function relationships within the same individuals to test whether structural changes (e.g. in gray matter volume or white-matter tract integrity) underpin the observed deficits in resting state activity.

An important limitation of the study is that, although the two groups did not differ in terms of years of education, IQ was not systematically measured or included as a covariate in the analyses. The CD group might have been expected to have lower IQs than the TD group [77], and it would have been optimal to control for this variable in the statistical analyses. Another limitation is that the participants recruited were all male adolescents, so it is not known whether these findings would generalize to females with CD or adults with antisocial personality disorder. Furthermore, the study did not use any instrument to assess personality traits associated with CD such as callous-unemotional (CU) traits [78]. Future resting state studies should assess personality traits such as CU traits to examine whether different subtypes of CD (i.e., those with limited prosocial emotions) or different symptom profiles map onto alterations in resting state fMRI activity. Finally, we note that the repetition time of the MRI sequence that we used is relatively long compared to other studies (3 s), which might introduce low-frequency noise to the resting-state signal. However, a low-pass filtering using a cut-off of 0.08 Hz was used in our study to control for the possible effects of physiological “noise” such as heart rate and respiration [79].

## Conclusion

In summary, the current study provides evidence supporting the hypothesis that CD is associated with dysfunction in emotion-related cortical regions, including the insula and amygdala, and regions involved in face processing such as the fusiform gyrus, lingual gyrus and left cuneus. The key contribution of this study is to show that these abnormalities in brain activity are observed even under resting conditions, and therefore might reflect changes in intrinsic baseline activity in CD. These findings might help to explain why individuals with CD show difficulties in emotion recognition and decision-making. It is also possible that group differences in baseline activity might have influenced some of the findings of earlier fMRI studies comparing CD and TD individuals using emotion-related or cognitive tasks.
